# Explaining biological differences between men and women by gendered mechanisms

**DOI:** 10.1186/s12982-023-00121-6

**Published:** 2023-03-23

**Authors:** Hélène Colineaux, Lola Neufcourt, Cyrille Delpierre, Michelle Kelly-Irving, Benoit Lepage

**Affiliations:** 1 EQUITY Team, CERPOP, INSERM, 37 Allees Jules Guesde, 31062 Toulouse, France; 2 Epidemiology Department, CHU Toulouse, 37 Allees Jules Guesde, 31062 Toulouse, France; 3 Biostatistic Department, Toulouse III University, 37 Allees Jules Guesde, 31062 Toulouse, France

**Keywords:** Sex, Gender, Embodiment, Allostatic load, Biomarker, Causal framework, Mediation analysis

## Abstract

**Background:**

The principal aim of this study was to explore if biological differences between men and women can be explained by gendered mechanisms.

**Methods:**

We used data from the 1958 National Child Development Study, including all the living subjects of the cohort at the outcome collection wave (44–45 years). We explored several biomarkers as outcomes: systolic blood pressure, triglycerides, LDL cholesterol, HbA1c, CRP, and cortisol. Three conceptualizations of gender have been used to define methodological strategies: (a) Gender as an individual characteristic; (b) Gender as an effect of sex on socio-behavioural characteristics; (c) Gender as an interaction between sex and the social environment, here the early-life social environment. We estimated the total effect of sex and the proportion of total effect of sex at birth eliminated by gender, measured by 3 different ways according to these 3 concepts, using g-computation.

**Results:**

The average level of each biomarker was significantly different according to sex at birth, higher in men for cardiometabolic biomarkers and higher in women for inflammatory and neuroendocrine biomarkers. The sizes of the differences were always smaller than one standard deviation but were larger than differences due to early-life deprivation, except for CRP. We observed gender mechanisms underlying these differences between men and women, even if the mediation effects were rarely statistically significant. These mechanisms were of three kinds: (1) mediation by socio-behavioural characteristics; (2) attenuation by gendered mechanisms; (3) interaction with early social environment. Indeed, we observed that being born into a deprived rather than non-deprived family increased metabolic and inflammatory biomarkers levels more strongly in females than in males.

**Conclusions:**

The biological differences between men and women seem to not be purely explained by biological mechanisms. The exploration of gender mechanisms opens new perspectives, in terms of methodology, understanding and potential applications.

**Supplementary Information:**

The online version contains supplementary material available at 10.1186/s12982-023-00121-6.

## Background

Observed differences between men and women* (see Table 1 for definitions of used terms*) in variables of a biological kind seem to be frequently attributed to mechanisms of biological kind (we will refer to them as sexed* mechanisms). These assumptions are sometimes explicitly expressed, for example in a course about “Sex and Gender in the Analysis of Data”, where haemoglobin, kidney function, height, lean body mass are described as “sex-related variables”, defined as “measurable biological or physiological parameters that systematically differ between men and women *due to* genetic or hormonal factors” [1]. Assuming sexed mechanisms can also be implicit, for example when sex-specific thresholds are set to control for presumed sexual dimorphism [2–4]. We even find this idea in the classic and often cited definition of sex and gender* in the biomedical literature, where “gender” refers to the social differences between men and women and “sex” refers to the biological differences between men and women [5], which can be understood as *all the* biological differences. While this simple definition may facilitate a broad understanding of ‘*what is sex’* versus ‘*what is gender’*, it also formalizes a false dichotomy where sex/ gender is understood as biological/ social.

**Table 1:** Definitions given to the used terms

Terms	Definitions
Men and women	Here, we use the term “men” or “women” in the sense of “male-born” and “female-born” individuals, without referring to gender identity or performance
Sex or sexed	We use the term "sexed" instead of “sex” to qualify *mechanisms* related to biological sexual dimorphism. For example, "sex differences" designates differences observed according to the sex assigned at birth, without hypotheses on their underlying biological or social mechanisms, whereas "sexed differences" would designate differences explained by biological mechanisms linked to sex
Gender	“Gender” is a complex concept and can refer to several phenomena. Especially in social sciences, it may refer to the process which polarises humanity and the characteristics of humanity into two categories "masculine" or "feminine", i.e., the binary and hierarchical categorisation system, including the phenomenon of domination relationship between the two categories [6]. The term of “gender” can also refer to an experience of self (gender personality or identity) [7, 8] In epidemiology, the term often seems to refer (1) either to the level at which the social characteristics and behaviours of an individual fits the stereotypes/ norms of masculinity or femininity; (2) or to the fact that, or the process by which, social characteristics and behaviours are differently distributed according to the binary sex at birth [9]. The gender concepts we used here are described in the "methods" section

However, social and biological phenomena are not so compartmentalized. Human life courses, environments, experiences, and behaviours shape our biology. Social life is biologically “embodied” [10]. Men and women live different life courses in our gendered binary world. Indeed, individuals, according to their sex assigned at birth, will not be subjected during their lives to the same physical, social, economic, cultural, and emotional exposures, they will not experience the same stressors, nor perceive and react to them in the same way, they will not adopt the same behaviours, etc. All these exposures that are distributed differently according to the sex assigned at birth will have different biological consequences in men and women. The gendered social structuring leads to the gendered construction of biology and health. Therefore, the biological and health differences observed between men and women could, at least in part, be explained by gender-based social mechanisms.

The concept of allostatic load emerged in the field of neurobiology, proposed by Mc Ewen to designate the cumulative multi-system physiological consequences of the repeated activation of adaptation (allostasis) processes by the organism in the face of the challenges it encounters during its existence [11]. This concept has been adopted by social epidemiology [12], since Teresa Seeman’s work [13], to explore how various social experiences—as adversity, discrimination, education, behaviours, etc. –, through stressful experiences and regulation, are biologically embodied [12]. In 1997, Seeman proposed the first epidemiological measurement of allostatic load [14], based on various biomarkers, which has since been widely used in the field of social epidemiology. These biomarkers measured the effect of experiences and regulations of stress in several physiological systems: the primary system (neuroendocrine), and, more often, secondary systems (cardiovascular, metabolic, inflammatory) [15]. Many of the biomarkers used to measure allostatic load have different distributions between men and women. These differences have been attributed to sexual dimorphisms [2–4], due to difference in sex hormones (e.g. oestrogen and testosterone) [16–18]. Yet socio-cultural factors and behaviours could also explain some of the differences in distribution [16, 19].

In this study, we aimed to explore whether differences in biomarkers observed between men and women are explained, at least partly, by three different gender mechanisms analysed using three different analytical strategies.

## Methods

### Data and population

We used data from the 1958 National Child Development Study (NCDS), one of the national British birth cohorts, which includes all people born during one week in 1958 (n = 18,555). Data on life conditions and experiences, about family, education, work, and health, were collected in twelve waves from birth to age 62 by the Centre for Longitudinal Studies. The NCDS has been described in detail elsewhere [20]. Detailed review of the ethical practices throughout NCDS is available at [https://cls.ucl.ac.uk/wp-content/uploads/2017/07/NCDS-Ethical-review-an-Consent-2014.pdf].

**Fig. 1:**
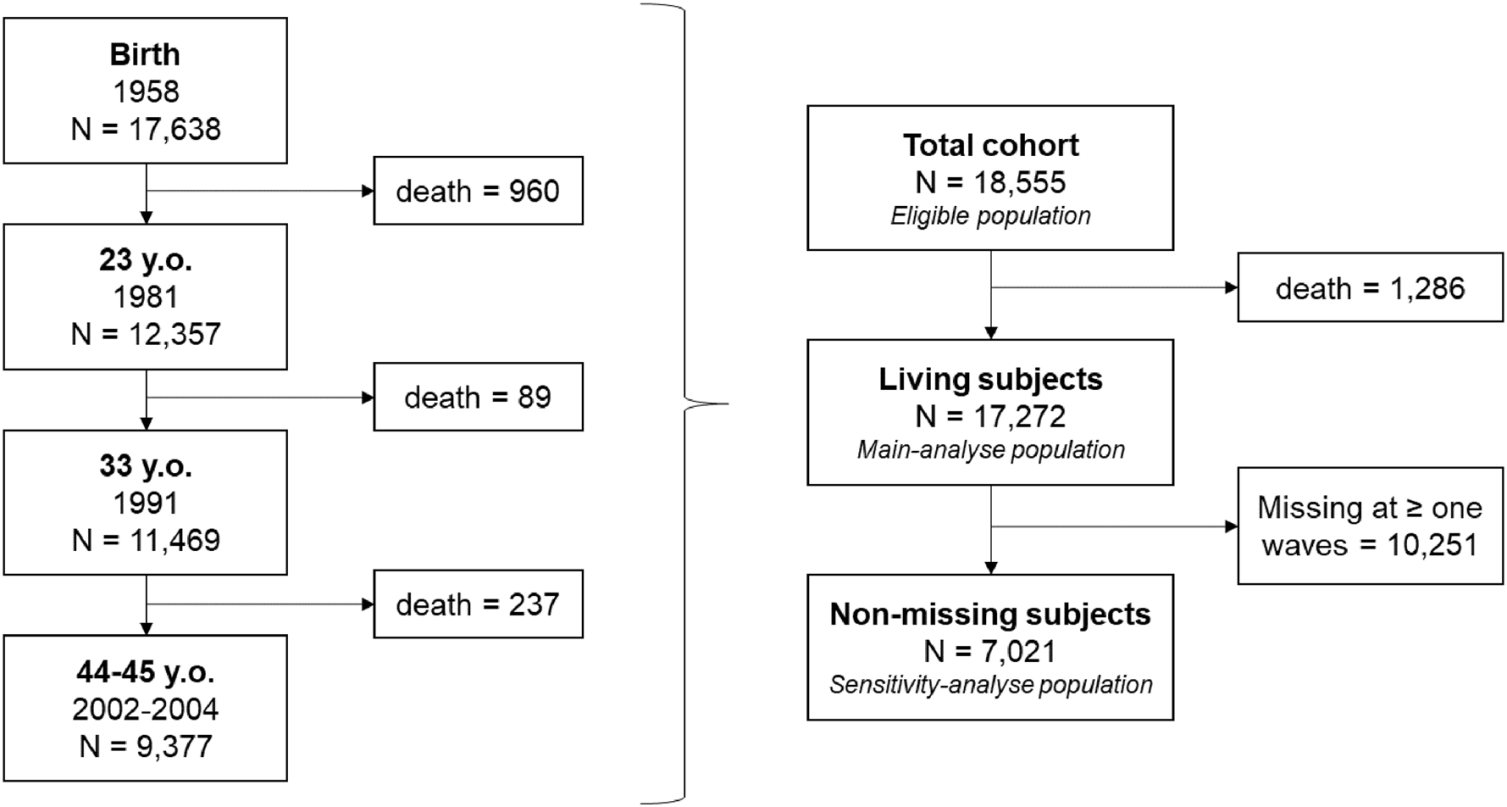
Flow chart

For this study, we used data collected during the first (1958, birth, N = 17,638), fourth (1981, 23 years, N = 12,357), fifth (1991, 33 years, N = 16,174) and biomedical waves (2002–2004, 44–45 years, N = 9,377). See the flow chart in Fig. 1. To reduce selection bias, we included all the living subjects at the time of the biomedical waves [21], when outcome variables had been collected (N = 17,272). Indeed, the total cohort is assumed to be representative of the generation, but the subjects are not missing at random at each wave. As a consequence, including only non-missing participants can leads to collider bias [22]. We therefore chose to include all living participants, to preserve the population structure, and imputed missing data. We however also performed a sensitivity analysis on participants who participated at the four used collection waves, involving more selection bias but fewer missing data (N = 7,021).

### Gender concepts

**Fig. 2:**
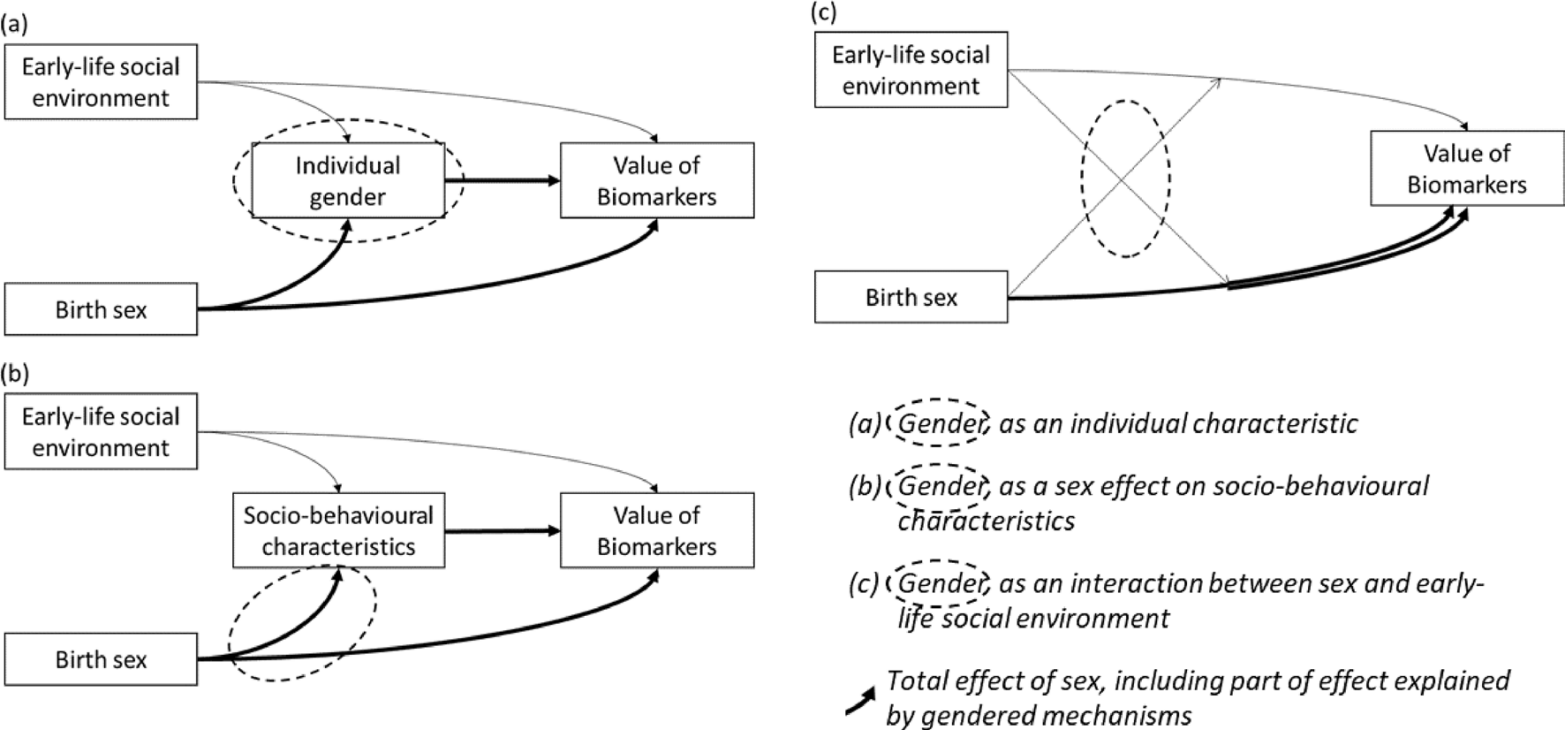
Conceptual graphs for three conceptualizations of gender

In this study, we explored *gender* as (1) the level at which the social characteristics and behaviours of an individual fits the stereotypes/ norms of masculinity or femininity (gender performance); and (2) the fact that, or the process by which, social characteristics and behaviours are differently distributed according to the binary sex at birth (gender pressure) [9]. These concepts can be operationalized in three ways within the epistemological and methodological framework of epidemiology, as detailed elsewhere [9] (see Fig. 2):

- *Gender as an individual characteristic*: gender refers to how an individual performs their gender, according to the norms of gender in the population in which they are socially active. This corresponds to the concept of gender performance. E.g., an individual can be said to have a “feminine” gender if they have mainly social characteristics considered as feminine, like having more care activities (childcare, looking after older people, nursing). This conceptualization implies understanding gender as an individually defined *variable*.
- *Gender as an effect of sex on socio-behavioural characteristics*: gender refers to the fact that socio-behavioural characteristics are differently distributed according to the sex at birth. This corresponds to gender as a gender pressure [9]. E.g., gender refers to the systemic process by which women are more likely to engage in caregiving activities than men in a given population. This conceptualization involves understanding gender as an *effect* of sex on one or more social-behavioural characteristics.
- *Gender as an interaction between sex and the early-life social environment*: if sex differences are not stable between social groups, we can explain these sex differences by gender mechanisms [9]. This third way of thinking about gender also refers to gender as a gender pressure as in conceptualization (b), but it takes into account the fact that the systemic process of gender varies, in its form or intensity, across social groups. E.g., if care activities are more often found in women in population A but in men in population B, we can conclude that the fact that care attitudes are associated with a sex is not “natural” but linked to systemic gender mechanisms. This is symmetrically equivalent to the fact that a given social environment does not have the same effect, through socialisation, on an individual, depending on their sex attributed at birth [9]. This conceptualisation involves understanding gender as a *difference in effects*, i.e. an interaction. In theory, this effect can concern the whole social environment, at any age, but to simplify the approach, we here considered only the early-life social environment, which is a priori independent of the sex at birth. This third conceptualisation can be seen as part of an intersectional approach to gender [23].

Here, we did not address gender as an experience of self (gender identity) or as a given kind of psyche (gender personality).

The conceptualizations of gender imply specific analytical strategies to meet the objective of identifying gender mechanisms to explain sex differences in biomarkers (See “Analyses” section). We refer to the corresponding strategy by the letter for each corresponding conceptualization (a, b, c).

### Measures

#### Outcomes: biomarkers

When individuals were about 45 years old, biomedical data were collected through a survey and a home-based clinical assessment (blood, saliva samples and anthropometric measurements) [24]. We explored several of these biomarkers, representing the four most frequent systems used to construct the score of allostatic load [25]: systolic blood pressure (SBP) for the cardiovascular system; triglycerides, low density lipoprotein (LDL) cholesterol and haemoglobin A1c (HbA1c) for the metabolic system; C-reactive protein (CRP) for the inflammatory system; and cortisol for the neuroendocrine system. When the distribution was too asymmetric, the variables were log transformed (triglycerides, CRP and cortisol).

#### Exposures: sex and early social environment

As our main exposure measure, we used sex attributed at birth. Relative to the effect of sex on the outcome, the early-life social environment was a competitive exposure and a confounder of the mediator-outcome relationship in strategies (a) and (b). In strategy (c), the early-life social environment was a modifier of the effect of sex on the outcome (see Fig. 2). We used two variables to characterise the early-life social environment at the time of the cohort member’s birth: educational level of the cohort member’s mother (school leaving age of 15 versus stayed at school beyond age 15, i.e., “O level”) and their other parent’s (or mother’s partner) social class (manual or non-manual social class). We used these variables to define two groups: the deprived group if the mother had a short education and the other parent a manual social class, and the non-deprived group in all other cases.

#### Mediators: gender scores and socio-behavioural characteristics

In strategies (a) and (b), we explore gender processes through mediator(s) (see Fig. 2). In strategy (a) gender was conceptualised as an individual characteristic which was measured by a gender score based on socio-behavioural variables. The mediator was this score. In strategy (b), gender was conceptualised as a sex effect on socio-behavioural characteristics. In this approach, mediators were the same socio-behavioural variables than those used to compute the gender score but kept separated.

##### Choice of socio-behavioural characteristics

In each strategy, we used the same set of socio-behavioural variables, either to compute the score (a) or separately (b). We consider that gender processes do not simply impact aspects of life classically described as gendered (like domestic load, type of occupation, etc.) but diffuses into all socio-behavioural dimensions. We had therefore chosen to use a larger set of socio-behavioural individual characteristics for which we assumed a priori to be distributed differently according to sex, because of the gender processes, and which may a priori have an impact on biomarkers and health.

Gender processes are multi-level, multidimensional and highly diffuse [7], so much so that we could say that every aspect of a human's life is impacted by the gender norms of the society in which they live. It impacts their identity (“how an individual sees themselves”), their roles (behaviours, experiences, expectations), their relations (“how individuals interact with and are treated by others”) and their relative power in different institutions (“political, educational, religious, media, medical, cultural and social institutions”) [7]. It seems impossible to capture all the aspects of life impacted by gender phenomenon [9]. We therefore sought to characterize, as broadly as possible, various dimensions of social life from the data available in the cohort.

Individual social characteristics that have an impact on health are equally multi-level, multi-dimensional and diffuse. They can be classified in two types: behaviours and social advantages/ disadvantages, i.e., resources which give the individual a varying degree of control and resilience over their environment, their experiences, and their life course. According to Bourdieu, these advantages/ disadvantages can be categorised into three dimensions: cultural capital, economic capital and social capital [26].

We therefore used several variables to characterise the three dimensions of capital and the behaviours. We made the a priori hypothesis that the distributions of these variables may vary according to sex, due to the gender processes:

- *Cultural capital* refers to knowledge, skills and integrated attitudes that will influence the way an individual sees the world, thinks, behaves, lives and acts [26]. In this study, we have represented these resources through five measures: “educational level at 23” (more or less than O level), “literacy at 23” (declared difficulties or not), “numeracy at 23” (declared difficulties or not), “often reads books at 23” (at least once a month) and “driver’s license at 33”. The driver's license is not a classic criterion of cultural capital, but we considered that it corresponded to the definition of a skill giving an increased control on the environment, an increased capacity to act in social life.
- *Economic capital* refers to the material and financial resources of individuals and the means to produce them [26]. In this study, we measured economic reserves with “personal savings at 23” and qualified the resources to produce them through “paid work at 33” and “social class at 33” (manual or non-manual).
- *Social capital* refers to an individual's social network, its size, value, and the degree of usefulness of these relationships [26]. In this study, we used the frequency of friends’ visits at 23 (more or less than once a week) as a marker of social support and “being religious at 23” as a marker of belonging to a community. The three variables “child(ren) at 23”, “married at 23”, and “doing laundry at 33” are markers of an affective and family support, but also of the domestic burden, counterpart of this resource.
- To characterize *behaviours*, we chose behavioural variables which can be considered as protective or a risk for health: “smoking (≥ 1 cigarette/day)” at 23 and 33, “everyday alcohol drinking” at 23 and 33, “frequent fried food” at 33 (more than once a week), “sport” at 23 and 33 (at least once a month). Risk taking being "a value and reality associated with masculinity" and which penalizes men by "causing them to perish" [27], we also used a proxy of risk-taking behaviours with the variable “have attended hospital or casualty department for any kind of accident or assault between 23 and 33”.

##### Impact of the choice of variables

The choice of variables to explore the gender phenomenon is largely based on their availability in the database and would have varied widely if another cohort had been used. To explore the impact of the variables availability on results, we constituted three different sets of variables and performed the analyses, for the strategies (a) and (b), with these different sets, "as if", in each case, we had only these variables to characterize the same phenomenon of interest. The sets were:

- *Complete set*: all the listed-above variables had been used. It was the main analysis.
- *Behavioural set*: only the behavioural variables had been used, *as if* only these variables were available.
- *Small set*: only 4 social characteristics (educational level at 23, social class at 33, frequency of friends’ visits at 23, and marital status at 23) and 4 variables for behaviours (sport, diet, smoking and alcohol at 33), *as if* only these variables were available.

##### Scores computation

In strategy (a), we wanted to capture gender as an individual characteristic, corresponding to the level at which the social characteristics and behaviours of an individual meets the standards of masculinity or femininity. To measure the gender corresponding to this definition, we used the "gender diagnosis" method [28–30]. The gender score produced by this method corresponds to a measure of the level at which an individual complies with a set of elements constituting femininity or masculinity in a given population, place and time, i.e., as the probability of being "predicted male or female" from social dimensions [9].

To construct the score, we modelled sex at birth by socio-behavioural characteristics using logistic regression, for each set of variables defined above. The gender score corresponded to the predicted probability by the model of sex at birth, a continuum from 0 “predict female by their socio-behavioural characteristics”, proxy of “gendered in a feminine way”, to 1 “predict male by their socio-behavioural characteristics”, proxy of “gendered in a masculine way”.

### Analyses

All the analyses were performed with R release 4.1.3, with the Tidyverse packages. To deal with missing data, we performed a single stochastic imputation using the MICE package in R [31] on each of 1,000 bootstrapped databases, also used to computed 95% confidence intervals [32, 33].

#### Descriptive analyses

We first described the exposures, mediators, and outcomes in excluded participants (dead at the biomedical waves) and included subjects (living at the biomedical wave), with number of missing data, and number and percentage of the variable categories, or mean and standard for quantitative variables. We also described these variables in the imputed bootstrapped databases, with mean of percentages or means and confidence intervals (2.5 and 97.5 percentiles), computed on 1,000 bootstrapped imputed datasets. The results of this description are given in Additional file 1. We then described socio-behavioural characteristics and gender scores by sex, with mean value and confidence intervals (2.5 and 97.5 percentiles) of percentages or means, computed on 1,000 bootstrapped imputed datasets.

#### Causal analyses

The mean value of each biomarker at 44–45 years of age was estimated under several counterfactual scenarios that differed by exposure and/ or mediator assignment. The notation ${\mathbb{E}}\left({Y}_{A=a}\right)$ represents the expected potential outcome (mean of Y) in the counterfactual scenario in which the exposure is set to $A = a$. Under the randomization assumption (no residual confounding) and the consistency assumption (effect of $A$ is the same whether observed or given by intervention), ${\mathbb{E}}({Y}_{A=a}$) was estimated using g-computation. Linear regressions were used to estimate conditional expectations of the outcome, denoted $\overline{Q }\left(A,L\right)= {\mathbb{E}}(Y|A,L)$, with $L$, the confounders. From the estimated $\overline{Q }\left(A,L\right)$ functions, we predicted the value of biomarker $Y$ for each member $i$ under the counterfactual scenarios. Target causal parameters (estimands), described below, were defined in an additive scale as the difference between the mean of potential outcomes in two scenarios.

##### Total effect of sex

We first aimed to measure the size of sex-differences in biomarker. The estimands were the total effect $TE$ of sex at birth $S$ on each biomarker $Y$, defined as the difference between the mean outcome had all the population been born male and the mean outcome had all the population been born female, denoted:

$$TE= {\mathbb{E}}[{Y}_{S=male }-{Y}_{S=female}]$$

In the $\overline{Q }\left(S,E\right)$ functions used to estimate the potential outcomes, $E$ contained the early-life social environment variable. We included an interaction term between $S$ and $E$. The outcomes were first used in their original scale, the $TE$ is therefore expressed in these units of measure, e.g., in mmHg for systolic blood pressure. They were then standardized as: $z=\frac{y- \mu }{\sigma }$ where $\mu$ and $\sigma$ are the mean and standard deviation of the outcome in each imputed bootstrapped data set. The $TE$ is therefore expressed in standard deviation, e.g., a total effect of 1 corresponds to a mean difference of 1 standard deviation between men and women.

##### Strategy (a): mediation by a gender score

The principal objective of the study was to identify gender mechanisms that explain sex-differences in biomarkers. With gender conceptualized as an individual characteristic (a), the estimand corresponded to the proportion of the total effect of sex on biomarkers which disappear when all the individuals are gendered in the same way, i.e., the eliminated proportion by gender score $G$, denoted ${EP}^{G}$. The eliminated proportion ${EP}^{G}$ was measured as the difference between the total effect of sex and the remaining effect of sex when all the population is gendered in the same way (gender score fixed at 0.5), divided by the total effect of sex [34]. The remaining effect of sex when all the population is gendered in the same way corresponds to the controlled direct effect ${CDE}^{G}$, which was defined here as the difference between the mean outcome had all the population been born male and the mediator (gender score) set at a given value (here 0.5) and the mean outcome had all the population been born female and the mediator (gender score) set at the same value (0.5) [35]. In the $\overline{Q }\left(S,E\right)$ and $\overline{Q }\left(S,G,E\right)$ functions used to estimate the potential outcomes, $E$ contained the early-life social environment variable (mediator-outcome confounder). We included an interaction term between $S$ and $E$, but not with $G$. Finally, we had:

$${CDE}^{G}= {\mathbb{E}}[{Y}_{S=male, G=0.5}-{Y}_{S=female,G=0.5}]$$ and $${EP}^{G}=\frac{TE-{CDE}^{G}}{TE}$$

##### Strategy (b): mediation by social characteristics

With gender conceptualized as an effect of sex on socio-behavioural characteristics (b), the estimand corresponded to the proportion of the total effect of sex on biomarkers which disappears when all the individuals have the same socio-behavioural characteristics, i.e., the eliminated proportion by the set of socio-behavioural characteristics $\Sigma$, denoted ${EP}^{\Sigma }$. The eliminated proportion ${EP}^{\Sigma }$ was measured as the difference between the total effect of sex and the remaining effect of sex when all the population has the same socio-behavioural characteristics (see Additional file 1 for detailed fixed values ${\varepsilon }^{*}$ for each variables), divided by the total effect of sex [34]. The remaining effect when all the population has the same socio-behavioural characteristics corresponds to the controlled direct effect ${CDE}^{\Sigma }$, which was defined here as the difference between the mean outcome had all the population been born male and all the socio-behavioural characteristics set at a given value (see Additional file 1) and the mean outcome had all the population been born female and all the socio-behavioural characteristics set at the same value [35]. In the $\overline{Q }\left(S,E\right)$ and $\overline{Q }\left(S,\Sigma ,E\right)$ functions used to estimate the potential outcomes, $E$ contained the early-life social environment variable. We included an interaction term between $S$ and $E$, but not with $\Sigma$. We therefore had:

$${CDE}^{\Sigma }= {\mathbb{E}}[{Y}_{S=male,\Sigma ={\varepsilon }^{*}}-{Y}_{S=female,\Sigma ={\varepsilon }^{*}}]$$ and $${EP}^{\Sigma }=\frac{TE-{CDE}^{\Sigma }}{TE}$$

##### Strategy (c): considering an interaction between sex and the early-life social environment

With gender conceptualized as an interaction between the sex at birth and the social environment (c), the estimand corresponded to the proportion of the total effect of sex on biomarkers which disappears when all the individuals have a non-gendered social environment. We considered that an observed non-gendered social environment was not realistic, so we rather considered a “less-gendered environment”. Here, to define the social environment, we considered only the early-life social environment $E$. We made the a priori hypothesis that the non-deprived group was less gendered than the deprived group. Therefore, the eliminated proportion ${EP}^{\mathrm{E}}$ was measured as the difference between the total effect of sex and the remaining effect of sex when all the population is exposed to a non-deprived early-life social environment $E = 0$, divided by the total effect of sex. The remaining effect when all the population is exposed to a non-deprived early-life social environment corresponds to the total effect of sex when $E=0$, denoted ${TE}^{0}$ and defined as the difference between the mean outcome had all the population been born male and the early-life social environment been non-deprived, and the mean outcome had all the population been born female and the early-life social environment been non-deprived. The model $\overline{Q }\left(S,E\right)$ used to estimate potential outcomes under these scenarios considered an interaction term between $S$ and $E$. We finally had:

$${TE}^{0} = {\mathbb{E}}[{Y}_{S=male ,E=0}-{Y}_{S=female,E=0}]$$ and $${EP}^{\mathrm{L}}=\frac{TE-{TE}^{0} }{TE}$$

##### Complementary analyses regarding interactions

We also present in the results section a more detailed description of the interaction effects, with mean value and confidence intervals (2.5 and 97.5 percentiles) of means, computed on 1,000 bootstrapped imputed datasets, for each biomarker in each category of sex and early-life social environment. We also computed the total effect of sex in each stratum of early social environment and total effect of early social environment in each stratum of sex. We finally estimated the interaction effect $IE$ of sex and early social environment, defined as:

$$IE = \,{\mathbb{E}}[{Y}_{S=male E=1}-{Y}_{S=male ,E=0}-{Y}_{S=female,E=1}+{Y}_{S=female,E=0}]$$

## Results

### Description of population

We included the 17,272 participants alive at the biomedical waves, 51% of whom were born male (see Additional file 1 for detailed description, Table a). Within our sample, 75% had a short-educated mother at their birth, and for 73%, the other parent was from manual social class.

The 1,286 participants who died before the biomedical survey at 44 years (see Additional file 1: Table a) were more often men (59%) and more often socially deprived (80% had a short-educated mother at their birth and for 78%, the other parent was from manual social class). The sensitivity analysis population—those with complete data for all the waves—(see Additional file 1: Table d) is globally more advantaged and with fewer men (48%) than in the main analysis population.

Most of the analysed variables of social resources, experiences, and behaviours at 23 and 33 were differently distributed according to sex at birth (see Table 2). In this specific population, on average, male-born and female-born individuals did not have the same type of cultural capital: a higher percentage of women than men had a level of education above O-level, declared fewer literacy difficulties and read more frequently than men, while a higher percentage of men had a driving licence than women. On average, economic capital also differed: a higher percentage of women had a non-manual social class at 33 compared to men, whereas men had a higher amount of savings at 23 and had more frequently a paid job at 23. Social capital was higher among women: a higher proportion of them were married and with children at age 23, reported being part of a religious community and saw their friends more frequently. As a counterpart, they were also much more likely to carry the domestic load, measured here by the laundry load. Regarding behaviours, fatty diets, risk-taking behaviours, regular alcohol consumption and smoking were more common in individuals born male, as the fact of being physically active at age 23.

**Table 2:** Distribution of social characteristics at 23 and 33 by sex, NCDS-58 cohort (N = 17,272)

	Male-born			Female-born			M—F
	%	95%CI		%	95%CI		%
**Cultural capital**							
Less than O level at 23	42.6	[41.2 to 43.9]		35.9	[34.6 to 37.2]		+ 6.7
No numeracy problems at 23	94.8	[94.1 to 95.4]		94.5	[93.8 to 95.1]		+ 0.3
Literacy problems at 23	12.6	[11.6 to 13.5]		7.0	[6.3 to 7.8]		+ 5.6
Does not often read at 23	45.6	[44.2 to 46.9]		32.6	[31.2 to 34.0]		+ 13.0
Driver’s license at 33	92.5	[91.7 to 93.3]		80.9	[79.6 to 82.1]		+ 11.6
**Economic capital**							
Personal savings > median at 23	54.5	[53.0 to 56.0]		45.7	[44.4 to 47.2]		+ 8.8
Paid work at 23	90.1	[89.1 to 91.0]		68.5	[67.2 to 69.9]		+ 21.6
Manual social class at 33	50.8	[49.3 to 52.4]		33.4	[32.0 to 34.9]		+ 17.4
**Social capital**							
Does not often see friend at 23	35.6	[34.1 to 37.0]		27.6	[26.4 to 28.8]		+ 8.0
Not married at 23	65.2	[63.9 to 66.5]		45.7	[44.4 to 47.1]		+ 19.5
No child at 23	82.0	[80.9 to 83.1]		66.6	[65.3 to 67.8]		+ 15.4
Does not do laundry at 33	86.0	[84.7 to 87.3]		4.2	[3.5 to 4.9]		+ 81.8
Not religious at 23	49.6	[48.2 to 51.1]		32.5	[31.2 to 33.8]		+ 17.1
**Behaviours**							
Smoking at 23	39.9	[38.6 to 41.2]		38.3	[36.9 to 39.5]		+ 1.6
Smoking at 33	32.6	[31.3 to 34.0]		32.0	[30.5 to 33.4]		+ 0.6
Alcohol every day at 23	31.4	[30.1 to 32.7]		9.8	[9.0 to 10.6]		+ 21.6
Alcohol every day at 33	17.6	[16.4 to 18.7]		7.0	[6.4 to 7.8]		+ 10.6
Often eats fried food at 33	56.6	[55.0 to 58.2]		35.0	[33.7 to 36.5]		+ 21.6
Often practices sport at 23	59.3	[57.8 to 60.7]		35.8	[34.5 to 37.1]		+ 23.5
Does not practice sport at 33	77.8	[76.4 to 79.0]		77.4	[76.1 to 78.7]		+ 0.4
Accident between 23 and 33	58.3	[56.8 to 59.8]		24.8	[23.5 to 26.0]		+ 33.5
**Gender scores, total population**							
Complete set*	0.87	[0.85 to 0.88]		0.13	[0.12 to 0.14]		+ 0.74
Behavioural set*	0.61	[0.60 to 0.62]		0.37	[0.36 to 0.38]		+ 0.24
Small set*	0.56	[0.55 to 0.57]		0.42	[0.41 to 0.43]		+ 0.14
**Gender scores, in deprived-born group**							
Complete set*	0.87	[0.86 to 0.88]		0.12	[0.11 to 0.13]		+ 0.75
Behavioural set*	0.62	[0.61 to 0.64]		0.37	[0.36 to 0.38]		+ 0.25
Small set*	0.55	[0.55 to 0.57]		0.41	[0.40 to 0.42]		+ 0.14
**Gender scores, in advantaged-born group**							
Complete set*	0.85	[0.84 to 0.87]		0.14	[0.13 to 0.16]		+ 0.71
Behavioural set*	0.59	[0.58 to 0.60]		0.37	[0.36 to 0.38]		+ 0.22
Small set*	0.57	[0.56 to 0.58]		0.43	[0.42 to 0.45]		+ 0.14

All categorical variables were binary. Showing variable categories that were the most frequent among male-born participants

“95%CI” corresponds to the confidence intervals computed on 1,000 bootstrapped imputed datasets

“M-F” corresponds to the male to female differences of observed probabilities (%)

* = quantitative variables, mean and 95%CI of mean are given

Regarding the gender scores, the mean was 0.13 (sd = 0.19) in female-born individuals and 0.87 (sd = 0.24) in male-born participants (complete set). The higher the number of variables used to estimate the score, the more discriminating the score. Groups defined by the early-life social environment were slightly differently gendered. Results confirmed the a priori hypothesis that a non-deprived early-life social environment was in a certain way less gendered than deprived early-life social environment, as the sex-gap was (slightly) smaller in the non-deprived group.

The sensitivity analysis performed only on participants who attended all 4 waves of data collection (N = 7021) showed similar distributions (see Additional file 1 Table e).

### Total effect of sex on biomarkers

The distributions of all the analysed biomarkers were significantly different according to sex at birth (see Table 3). Cardiometabolic biomarkers were all on average higher for male-born individuals, whereas inflammatory and endocrine biomarkers were on average higher for female-born individuals. For example, the systolic blood pressure at 44–45 years old was on average 12.45 mmHg (95CI = [11.77 to 13.18]) higher in individuals born male than in those born female. However, the size of differences varied and was always smaller than 1 standard deviation (sd), from − 0.07 [− 0.14 to − 0.01] standard deviation for logarithm of cortisol, to + 0.75 [0.71 to 0.79] standard deviation for systolic blood pressure.

**Table 3:** Total effect (TE) of being born male on biomarkers at 44–45, NCDS-58 cohort (N = 17,272)

	**Female**		**Original scale**			**Z-scores**	
	Mean (sd)		TE	95%CI		TE	95%CI
Systolic Blood Pressure (mmHg)	120.5 (15.5)		+ 12.45	[11.77 to 13.18]		+ 0.75	[0.71 to 0.79]
Log (Triglycerides (g/L))	0.32 (0.5)		+ 0.42	[0.39 to 0.45]		+ 0.70	[0.65 to 0.75]
LDL Cholesterol (mmol/L)	3.30 (0.9)		+ 0.31	[0.25 to 0.37]		+ 0.33	[0.27 to 0.39]
HbA1c (%)	5.21 (0.6)		+ 0.13	[0.09 to 0.16]		+ 0.17	[0.13 to 0.22]
Log (CRP (mg/L))	0.13 (1.3)		− 0.13	[− 0.2 to − 0.05]		− 0.11	[− 0.17 to − 0.04]
Log (Cortisol (µg))	2.94 (0.5)		− 0.04	[− 0.08 to − 0.01]		− 0.07	[− 0.14 to − 0.01]

*TE* = *total effect of being born *male* rather than female; 95%CI* = *bootstrapped confidence intervals (N* = *1,000)*

In comparison, being born into a deprived family was associated with increased levels of all biomarkers from + 0.04 sd [− 0.02 to 0.10] for cortisol to + 0.20 sd [0.13 to 0.26] for CRP (see Additional file 1: Table c).

The sensitivity analysis performed only on participants who attended the 4 waves of data collection (N = 7021) yielded similar conclusions (see Additional file 1: Tables f and g)

### Explained proportion of sex effect

Strategies (a) and (b) provided very similar results (see Table 4). According to the set of variables used to estimate the eliminated proportion, results varied. We describe results of strategies (a) and (b) in this section and results of strategy (c) in the following section.

**Table 4:** Eliminated proportion (EP) of sex effect, NCDS-58 cohort (N = 17,272)

	(a) By gender-score mediator			(b) By socio-behavioural mediators			(c) By early social environment		
	EP (%**)**	95%CI		EP (%)	95%CI		EP (%)	95%CI	
**Systolic Blood Pressure**							− 1.86	[− 6.7 to 3.0]	
Complete set	9.31	[− 2.6 to 21.4]		9.15	[− 3.2 to 21.1]				
Behavioural set	2.12	[− 1.7 to 5.9]		2.04	[− 1.9 to 5.9]				
Small set	4.38	[1.5 to 6.9]^*^		4.32	[1.4 to 6.9]^*^				
**Log (Triglycerides)**							− 7.67	[− 13.2 to − 2.1]^*^	
Complete set	− 2.51	[− 18.7 to 12.4]		− 5.52	[− 22.0 to 10.2]				
Behavioural set	− 0.22	[− 5.0 to 4.6]		− 0.35	[− 5.3 to 4.4]				
Small set	1.32	[− 2.1 to 4.9]		1.43	[− 2.0 to 5.1]				
**LDL Cholesterol**							− 14.28	[− 29.0 to − 1.8]^*^	
Complete set	2.75	[− 28.6 to 33.5]		− 8.87	[− 42.5 to 23.4]				
Behavioural set	− 0.03	[− 10.1 to 10.0]		0.01	[− 9.9 to 10.1]				
Small set	− 1.90	[− 9.4 to 5.7]		− 1.69	[− 9.3 to 5.8]				
**HbA1c**							− 4.95	[− 28.1 to 17.0]	
Complete set	− 7.88	[− 71.6 to 50.0]		− 32.73	[− 98.2 to 29.4]				
Behavioural set	− 13.20	[− 31.6 to 4.9]		− 14.60	[− 32.5 to 3.7]				
Small set	6.27	[− 7.6 to 21.9]		6.24	[− 7.4 to 21.7]				
**Log (CRP)**							63.34	[23.2 to 170.0]^*^	
Complete set	− 20.31	[− 158.4 to 103.2]		13.93	[− 121.5 to 160.8]				
Behavioural set	0.53	[− 43.4 to 38.5]		1.24	[− 42.5 to 38.8]				
Small set	− 16.45	[− 63.3 to 11.9]		− 16.33	[− 64.0 to 13.1]				
**Log (Cortisol)**							− 8.46	[− 121.3 to 71.1]	
Complete set	108.41	[− 143.5 to 562.0]		112.98	[− 136.4 to 545.9]				
Behavioural set	5.90	[− 72.8 to 99.3]		6.44	[− 70.2 to 97.7]				
Small set	0.64	[− 70.3 to 69.1]		1.24	[− 69.8 to 68.7]				

EP = Eliminated proportion; 95%CI = bootstrapped confidence intervals (N = 1000); * = 95%CI does not include zero

*Cardiovascular biomarker*: Systolic blood pressure at 44 was on average higher in male-born individuals of this population. Setting the gender score to 0.5 (a) or the socio-behavioural characteristics at their values of reference (b), we eliminated up to 9.3% [− 2.6 to 21.4] of the total effect of sex.

*Metabolic biomarkers*: Triglycerides, LDL Cholesterol and HbA1c at 44 were on average higher in male-born individuals. Setting the gender score to 0.5 (a) or the socio-behavioural characteristics at their values of reference (b), the eliminated proportions of sex effect on lipids varied from − 9% to + 3% according to the considered set of variables and were never statistically significant. Regarding HbA1c, setting the gender score to 0.5 (a) or the socio-behavioural characteristics at their values of reference (b), the eliminated proportions of sex effect varied from − 33% to + 6% according to the considered set of variables and were never statistically significant.

*Inflammatory biomarker*: CRP at 44 was on average lower in male-born individuals. Setting the gender score to 0.5 (a) or the socio-behavioural characteristics at their values of reference (b), the eliminated proportions of sex effect varied from − 20% to + 14% according to the considered set of variables and were never statistically significant.

*Neuroendocrine biomarker*: Cortisol at 44 was on average lower in male-born individuals. Setting the gender score to 0.5 (a) or the socio-behavioural characteristics at their values of reference (b), the eliminated proportions of sex effect varied from + 1% to + 112% according to the considered set of variables and were never statistically significant.

The sensitivity analysis performed only on participants who attended the 4 waves of data collection (N = 7021) yielded the same conclusions (see Additional file 1: Table h).

### Results regarding interaction

Results of strategy (c) was difficult to interpret only with the eliminated proportion. Moreover, the results seemed contradictory with the strategies (a) and (b) for several biomarkers. A more detailed description of interaction effects is given Table 5, with, for each biomarkers: mean values in each category of sex and early-life social environment (in original scale and as z-scores); total effect of sex in each stratum of early-life social environment; total effect of early-life social environment in each stratum of sex, additive interaction between sex and early-life social environment, as defined in the Methods section.

**Table 5:** Effects of sex and early-life social environment on biomarkers, NCDS-58 cohort (N = 17,272)

		Advantaged- born		Deprived-born		TE of early deprivation	
**SBP**	**Original scale**						
	Male born (mean)	132.0	[131.3 to 132.7]	133.6	[133.0 to 134.3]	+ 1.6	[0.7 to 2.5]
	Female born (mean)	119.3	[118.6 to 120.1]	121.3	[120.6 to 121.9]	+ 1.9	[1.0 to 2.9]
	TE of being born male	+ 12.7	[11.8 to 13.6]	+ 12.3	[11.5 to 13.2]	(− 0.38)	[− 1.38 to 0.60]
	**Z-score**						
	Male born (mean)	0.31	[0.27 to 0.35]	0.40	[0.38 to 0.43]	+ 0.09	[0.04 to 0.15]
	Female born (mean)	− 0.46	[− 0.5 to − 0.42]	− 0.34	[− 0.37 to − 0.31]	+ 0.12	[0.06 to 0.18]
	TE of being born male	+ 0.77	[0.72 to 0.82]	+ 0.74	[0.70 to 0.79]	(− 0.03)	[− 0.08 to 0.04]
**Log(triglycerides)**	**Original scale**						
	Male born (mean)	0.70	[0.67 to 0.73]	0.77	[0.74 to 0.80]	+ 0.07	[0.03 to 0.11]
	Female born (mean)	0.25	[0.22 to 0.28]	0.37	[0.34 to 0.40]	+ 0.12	[0.09 to 0.16]
	TE of being born male	+ 0.45	[0.41 to 0.49]	+ 0.40	[0.36 to 0.44]	(− 0.05)	[− 0.09 to − 0.01]
	**Z-score**						
	Male born (mean)	0.27	[0.22 to 0.32]	0.39	[0.35 to 0.42]	+ 0.12	[0.06 to 0.18]
	Female born (mean)	− 0.48	[− 0.53 to − 0.43]	− 0.28	[− 0.32 to − 0.24]	+ 0.20	[0.14 to 0.26]
	TE of being born male	+ 0.75	[0.69 to 0.82]	+ 0.67	[0.61 to 0.72]	(− 0.08)	[− 0.15 to − 0.02]
**LDL cholesterol**	**Original scale**						
	Male born (mean)	3.58	[3.52 to 3.63]	3.63	[3.57 to 3.70]	+ 0.05	[− 0.01 to 0.12]
	Female born (mean)	3.23	[3.18 to 3.28]	3.35	[3.30 to 3.40]	+ 0.12	[0.06 to 0.18]
	TE of being born male	+ 0.35	[0.29 to 0.42]	+ 0.28	[0.21 to 0.35]	(− 0.07)	[− 0.13 to − 0.01]
	**Z-score**						
	Male born (mean)	0.13	[0.08 to 0.17]	0.18	[0.14 to 0.22]	+ 0.05	[− 0.01 to 0.12]
	Female born (mean)	− 0.25	[− 0.29 to − 0.20]	− 0.12	[− 0.16 to − 0.07]	+ 0.13	[0.07 to 0.19]
	TE of being born male	+ 0.37	[0.31 to 0.44]	+ 0.30	[0.23 to 0.37]	(− 0.07)	[− 0.14 to − 0.01]
**HbA1c**	**Original scale**						
	Male born (mean)	5.29	[5.26 to 5.33]	5.36	[5.33 to 5.40]	+ 0.07	[0.03 to 0.12]
	Female born (mean)	5.16	[5.14 to 5.19]	5.24	[5.21 to 5.28]	+ 0.08	[0.05 to 0.12]
	TE of being born male	+ 0.13	[0.09 to 0.17]	+ 0.12	[0.08 to 0.16]	(− 0.01)	[− 0.05 to 0.04]
	**Z-score**						
	Male born (mean)	0.02	[− 0.02 to 0.07]	0.12	[0.09 to 0.16]	+ 0.10	[0.04 to 0.16]
	Female born (mean)	− 0.16	[− 0.19 to − 0.12]	− 0.04	[− 0.08 to − 0.01]	+ 0.11	[0.07 to 0.15]
	TE of being born male	+ 0.18	[0.12 to 0.23]	+ 0.17	[0.11 to 0.22]	(− 0.01)	[− 0.07 to 0.05]
**Log (CRP)**	**Original scale**						
	Male born (mean)	− 0.11	[− 0.18 to − 0.03]	0.07	[0.01 to 0.13]	+ 0.17	[0.09 to 0.26]
	Female born (mean)	− 0.06	[− 0.13 to 0.02]	0.25	[0.18 to 0.32]	+ 0.31	[0.22 to 0.4]
	TE of being born male	− 0.05	[− 0.13 to 0.04]	− 0.18	[− 0.27 to − 0.09]	(− 0.13)	[− 0.21 to − 0.05]
	**Z-score**						
	Male born (mean)	− 0.14	[− 0.19 to − 0.08]	0.00	[− 0.04 to 0.05]	+ 0.14	[0.07 to 0.22]
	Female born (mean)	− 0.1	[− 0.16 to − 0.05]	0.15	[0.11 to 0.20]	+ 0.25	[0.18 to 0.33]
	TE of being born male	− 0.04	[− 0.11 to 0.03]	− 0.15	[− 0.22 to − 0.08]	(− 0.11)	[− 0.17 to − 0.04]
**Log (Cortisol)**	**Original scale**						
	Male born (mean)	2.89	[2.86 to 2.92]	2.91	[2.89 to 2.94]	+ 0.03	[− 0.01 to 0.06]
	Female born (mean)	2.93	[2.9 to 2.96]	2.95	[2.92 to 2.98]	+ 0.02	[− 0.02 to 0.06]
	TE of being born male	− 0.04	[− 0.08 to 0.00]	− 0.04	[− 0.07 to 0.00]	(+ 0.00)	[− 0.03 to 0.04]
	**Z-score**						
	Male born (mean)	− 0.06	[− 0.12 to − 0.01]	− 0.02	[− 0.06 to 0.02]	+ 0.05	[− 0.02 to 0.11]
	Female born (mean)	0.01	[− 0.03 to 0.07]	0.05	[0.01 to 0.09]	+ 0.04	[− 0.03 to 0.10]
	TE of being born male	− 0.08	[− 0.15 to − 0.01]	− 0.07	[− 0.14 to 0.00]	(+ 0.01)	[− 0.05 to 0.08]

*SBP = Systolic Blood Pressure; TE = Total effect; 95%CI = bootstrapped confidence intervals (N = 1,000); results in bold and in brackets are the measures of additive interaction* ${(Y}_{11}-{Y}_{10})-\left({Y}_{01}-{Y}_{00}\right)$

*Regarding cardiometabolic biomarkers*, we observed that, contrary to what was expected, the sex gaps were smaller (negative eliminated proportions) or tended to be observed in people deprived versus non-deprived in early life. The detailed results (see Table 5) showed that a deprived early-life social environment increased the level of the four cardiometabolic biomarkers at 44 years old and these effects on lipids were stronger in women. E.g., the total effect of being born deprived on LDL cholesterol was + 0.05 standard deviation [− 0.01 to 0.12] in men and + 0,13 standard deviation [0.07 to 0.19] in women; so, the sex gap was + 0.37 standard deviation [0.31 to 0.44] in non-deprived group against + 0.30 standard deviation [0.23 to 0.37] in deprived group.

In other words, being born male and being born deprived increased, on average, the level of triglycerides and LDL cholesterol, but the effect of being born deprived was stronger in women. So, symmetrically, the effect of being born male was smaller in the deprived group, which explained the negative eliminated proportions of the sex effect by being born into a non-deprived early social environment (see Table 4).

*Regarding the inflammatory biomarker*, the sex gap was reduced by almost 64% in born non-deprived group (see Table 4). Indeed, the total effect of sex was − 0.04 standard deviation [− 0.11 to 0.03] in the non-deprived group and − 0.15 standard deviation [− 0.22 to − 0.08] in the deprived group (see Table 5). For this biomarker too, the effect of early deprivation was stronger in women than in men: + 0.25 [0.18 to 0.33] versus + 0.14 [0.07 to 0.22] standard deviation.

*Regarding the neuroendocrine biomarker*, the sex effect was similar in each group of early social environment and, symmetrically, the early-deprivation effect was similar between men and women.

The sensitivity analysis performed only on participants who attended the 4 waves of data collection (N = 7021) yielded the same conclusions (see Additional file 1: Table i).

## Discussion

### Main results

The distribution of each biomarker was significantly different according to sex at birth, higher in men for cardiometabolic biomarkers and higher in women for inflammatory and neuroendocrine biomarkers. The sizes of the differences were always smaller than one standard deviation but were larger than differences due to early-life deprivation, except for CRP.

We observed gender mechanisms underlying these differences between men and women, even if the mediation effect was rarely statistically significant. These mechanisms were of three kinds: (1) mediation by socio-behavioural characteristics, e.g., for SBP, 4.3% of the observed sex differences significantly disappeared when socio-behavioural characteristics measured at 23 and 33 years or gender score were set; (2) attenuation by gendered mechanisms: e.g., for HbA1c, sex differences tended to be larger when the socio-behavioural characteristics at 23 and 33 years were set to a common level; (3) interaction with early social environment: for metabolic and inflammatory biomarkers; e.g., we observed that being born into a deprived rather than non-deprived family increased triglycerides, LDL cholesterol and CRP levels more strongly in females than in males.

### Comparison with literature

In our study, cardiometabolic biomarkers (systolic blood pressure (SBP), triglycerides, LDL cholesterol and HbA1c) were on average higher in male-born individuals. The differences in SBP were partly mediated by the studied socio-behavioural characteristics. For triglycerides and LDL cholesterol, the sex difference varied with social environment at birth, suggesting that the effect of deprivation is different according to sex at birth and therefore that levels of serum lipids are influenced by gender mechanisms. These results are consistent with prior knowledge: if innate biologic factors explained a part of the biomarkers levels variability [36] and sex hormone levels [37], lifestyle also plays an important role on the whole cardiovascular and metabolic system. Indeed, medications, smoking, alcohol consumption, diet, sedentary lifestyle, obesity, etc. are identified risk factors of dyslipidaemia [38–40], high blood pressure [41, 42] and diabetes [43, 44]. These behaviours varying by sex at birth, through gender processes, it seems expected that part of the sex differences can be explained by socio-behavioural mechanisms. Pelletier et al. also showed that risks of hypertension and diabetes varied by sex and that these effects were partially mediated by a gender score in a different population [30]. This also confirms that sex disparities are partly explained by gender mechanisms.

In our study, CRP levels were on average slightly higher in female-born individuals. In the mediation approaches, the sex difference tended to be larger when socio-behavioural characteristics or gender scores were set at the same value. But these effects were not stable nor statistically significant. However, the sex difference varied with early social environment: female-born individuals had a greater increase in CRP with deprivation than male-born individuals. Genetic factors would account for approximately 40 to 50% of the variance of CRP [45]. Besides, genes related to immunity have been identified on the X chromosome, and would explain some immune differences between men and women, including the higher immune reactivity and the higher risk of autoimmune pathologies in women [46]. Oestrogen levels, particularly during pregnancy or menopause, are also known to interfere with CRP [45]. However, independently or in interaction with genetic factors, the levels of inflammatory biomarkers are higher in cases of high body fat, obesity, diabetes, smoking and hypertension, lower in case of certain deficiencies and with alcohol consumption and vary with diet, physical activity, year of schooling and with the use of certain medications [45, 47–49]. These socio-behavioural determinants varying by sex at birth, through gender processes, it seems expected that part of the sex differences can be explained by socio-behavioural mechanisms.

In our study, cortisol levels were on average slightly higher in female-born individuals. In the mediation approaches, the sex-difference tended to disappear when socio-behavioural characteristics or gender scores (complete set) were set at the same value. But these effects were not stable nor statistically significant. The sex difference did not vary either with early social environment. Differences between men and women in cortisol responsiveness to stress have been extensively described [18]. In particular, cortisol levels vary when oestrogen levels vary (menstrual cycle, pregnancy contraceptive use) [50]. However, there are socio-behavioural factors involved in the variability of cortisol of cortisol levels: the number and intensity of stressful situations [2], the perception of stress [51, 52], the type of stressors and their context [13, 50, 53–55]. In our study, we were not able to explain the differences between men and women in cortisol levels by gender mechanisms, but the observed differences were very small. We assume that, although differences have been observed in experimental situations of acute stress, salivary cortisol seems to be rather insensitive to identify differences in the daily life of the general population. While cortisol is a central biomarker of the allostatic load theory, it is the only biomarker that does not increase significantly with early social disadvantage, contrary to what one might expect [56, 57].

These findings provide a better understanding of the gendered biological incorporation, as a pathway to explain the links described in the literature between sex/gender and mental and physical health [30, 58–60]

### Strengths and limits

In this study, we used a large and well-known cohort, with a prospective collection of social and health data. Studying this cohort however involves several limitations. First, a large part of participants was lost to follow-up, and those are more disadvantaged than others, which can lead to selection bias. We chose therefore to include all the living subject (no missing data for death status) at the time of outcome collection to limit these selection bias. Secondly, and as a consequence of including not-attending participants, some data are missing, especially concerning biomarkers. To deal with this, we have made single stochastic imputations on 1,000 bootstrapped databases. We also performed a sensitivity analysis only on participants who attended all the four waves of data collection that we used, which produced similar results.

Our approach remained focused on an individual-centred definition of gender. We have not addressed the issues of gender structural impact, sexism, discrimination affecting queer people, etc., which are of course part of the gender issue, and which certainly also impact biology and health. Within our specified scope, we had defined three methodological strategies to explore how gender could explain health differences between men and women. The use of several strategies, with several sets of variables, allowed us to capture different phenomena, explore the robustness of our analyses and reveal the advantages and disadvantages of these different approaches, as summarized in Table 6.

**Table 6:** How to capture Gender effect: advantages and disadvantages of three approaches

Approaches	Main advantages	Main disadvantages
(a) Gender as an individual characteristic	∙ Through a single score, allows the measurement of a total effect of gender	∙ Sensitive to the type and number of variables to compute the score ∙ Difficult to interpret and conceptually questionable
(b) Gender as a sex-differentiated distribution of socio-behavioural characteristics	∙ Less information lost ∙ Less difficult to interpret ∙ Different paths could be analysed in a second step	∙ Sensitive to the type and number of variables ∙ Gender total effect not directly measurable
(c) Gender as a differential effect of the social environment depending on the sex at birth	∙ More consistent with the systemic concept of gender	∙ Depends on the heterogeneity of gender processes between the social groups in the studied population ∙ Complexity of the choice of scale, the presentation of results and the interpretation ∙ Only early-life variables can be used with these method (cannot be mediators of sex or other methods would be needed)

We consider that the strategy (c) is more consistent with the systemic concept of gender, whose variation across social categories is inherent. Intersectionality is a term proposed in the 1980s by the American law professor Kimberlé Crenshaw to refer to the fact that the relations of domination experienced by racialised women are not the same as those experienced by white women (Black Feminism movement) [61]. The term now refers more generally to the fact that the experiences of any individual are situated at the intersections of multiple categories, including gender, but also class, race, age, sexual orientation, ability, etc., which influence each other to create distinct experiences in different combinations [23]. In other words, the effect of these different categories do not add up but *"intersect in dynamic, complex, and surprising ways depending on context*" [23]. From an analytical point of view, the intersectional approach allows to question which variables are relevant to understanding a health phenomenon, but also how they interact with each other and how their effects vary over time or between populations [23]. This analytical framework has been used primarily in the social sciences, but many quantitative methods also allow for an intersectional analysis of a phenomenon in an intersectional way, such as interaction analyses [62, 63], which we applied here. However, in our study, the population seemed to be relatively homogeneous in term of gender processes, which reduces the relevance of the interaction approach with the early-life social deprivation here. The study of the intersection with ethnicity/ race/ migration might have been more sensitive, as it has been shown that the intersectional effect of these categories on health can be strong [58, 64], but this was not possible due to a small number of minoritized individuals in the study data. It will be interesting to replicate this analysis in other populations, as the dynamics of gender phenomena can vary extensively according to the generation, age, culture, context, etc., and the consequences on observed sex differences are probably also very diverse from one population to another.

In a previous work [9], we had proposed two strategies: one based on an individual approach of gender, measured by a gender score and the other based on an interaction analysis. Here we propose an additional approach (b), close to the strategy (a) but without a score. These two strategies lead to similar results. However, they do not have the same conceptual and methodological implications. First, the production of a score is, in our opinion, more at risk of over-interpretation of the results by forgetting the loss of information, simplification and non-exhaustiveness of an attempt to capture such a diffuse, complex, multilevel, intersectional phenomenon through one variable. It could also lead to an essentialization, and immobilization of what gender would be. In this sense, considering gender as being an *effect* of sex on socio-behavioural characteristics rather than directly composed by these characteristics, seems to us less risky. Secondly, the score complexifies the interpretation: it aims to capture the latent, diffuse phenomenon of gender performance that would have an impact on biology through various mechanisms which are not necessarily the variables used to calculate the score. Using these variables separately (b) implies, on the contrary, more causal, mechanistic hypotheses, i.e., that the effect may actually pass through each of the identified variables. This strategy renders assumptions and interpretations less obscure and gives more guidance for interventions [60]. Finally, the level of a gender score is not necessarily due to gender pressure alone, but also to other factors such as social class, age, generation, etc. [58]. The effect of this type of variable is therefore difficult to interpret as a strict gender effect, which would not include part of an effect of class, age, culture, etc. Again, the use of separate variables avoids over-interpretation of the results.

One of the central points of our work concerning the consideration of gender in epidemiology is to decentralise the problem from a question of gender measure, to propose a more dynamic and structural approach, focused on the strategy. We demonstrated that we can analyse the impact of gender without a gender variable, by conceptualising it as a structural phenomenon and operationalise it as an effect or a difference in effect.

### Perspectives

This study has identified gender mechanisms but has also opened many questions, which themselves raise complex methodological issues. First, it would seem interesting to explore more finely the paths of mediation involved. For example, we explained almost 10% of the sex-differences in SBP, but what exactly was this due to? To answer this, it would require making more precise hypotheses on the causal sequences between the different mediators in order to consider the intermediate confounding. Secondly, it would be potentially important to explore the interaction between sex and mediators. Indeed, the social characteristics, not only at birth but also across the life-course, probably does not have the same effect depending on the sex at birth. For example, having a paid job at 23 probably does not have the same impact on a woman's life than on a man's, as it might not have the same “value” socially. It would therefore be interesting to continue this work in order to consider not only other early-life social categories (material condition, social support, etc.), but also other social categories throughout the life course. The 4-way decomposition proposed by VanderWeele could allow us to explore these phenomena [65], but this must be analysed mediator by mediator. Finally, it would be interesting to combine the approaches of mediation and interaction in order to explore if we “captured” or not the same mechanisms with the mediation approach (strategy b) and the interaction approach (strategy c). These could be explored with a combination of the 4-way decomposition and a measure of interaction between two independent exposures [66].

An important implication of these results concerns the methods for calculating allostatic load. The methodological questions around the most appropriate way to measure allostatic load from biomarkers are diverse and still debated [4, 67]. The original and most widely used method is based on counting the number of biomarkers where the individual is "at risk", i.e., at one extreme of the distribution (often the highest or lowest quartile). When such data-driven thresholds are used to defined the “at-risk” groups, the question of whether these thresholds should be set according to sex or not is important, and unresolved [4]. Some authors justify a sex-specific threshold approach because they attribute differences in the average level of biomarkers to sexual dimorphisms which they want to control [2–4]. Many of the biomarkers used to measure allostatic load may indeed have physiologically different distributions between the sexes due to difference in sex hormones (e.g. oestrogen and testosterone) [16–18]. But socio-cultural factors and behaviours also explain some of the differences in distribution [16, 19], as the results of this study showed. Only few studies have looked at this issue and, if the method based on sex-specific thresholds seems to be more successful in terms of prediction [2, 4], the question of the methodological and conceptual relevance of this approach has not been asked. In general, dichotomisation should be avoid, mainly as it lead to an information loss [68]. But, beyond this point, a differentiated dichotomization is equivalent to controlling not only for the effect of sex but also to the effect of gender (as gender might explain some of the sex difference), on these variables. This can lead to gender bias, especially if the studied phenomena are social and gendered. We would, therefore, recommend using non-dichotomizing methods, such as the sum of z-scores, to calculate the allostatic load; and, at least, not to calculate a sex-specific score, but rather to adjust for sex in the model, including an interaction term between sex and allostatic load, to control for the effect of sexual dimorphism on biomarkers.

On the other hand, some sex differences are large, larger than a strong social determinant such as early-life deprivation. It was the case here for systolic blood pressure for example. However, clinical norms for this biomarker are not sex-differentiated, which may lead to underdiagnosis and therefore undertreatment in women, in this example.

## Conclusion

The biological differences between men and women seem not to be purely explained by sexed mechanisms. The exploration of gender mechanisms opens new perspectives, in terms of methodology, understanding and potential applications.

## Supplementary Information

**Additional file 1.** Additional tables.

## Data Availability

Datasets are available on request in https://cls.ucl.ac.uk/cls-studies/1958-national-child-development-study/. R scripts are available from the corresponding author on reasonable request.

## Abbreviations

NCDS: National Child Development Study
SBP: Systolic blood pressure;
LDL: Low density lipoprotein
HbA1c: Haemoglobin A1c
CRP: C-Reactive protein
TE: Total effect
EP: Eliminated proportion
CDE: Controlled direct effect
IE: Interaction effect
CI: Confidence interval
