# Supplementary material for: Explaining biological differences between men and women by gendered mechanisms
Source: Emerg Themes Epidemiol. 2023 Mar 23;20:2. doi: 10.1186/s12982-023-00121-6 (PMC10037796; doi:10.1186/s12982-023-00121-6)
Supplement: Supplementary file 1 — Additional file 1. Additional tables. [file 12982_2023_121_MOESM1_ESM.docx]

# Additional file

Table a. Description of populations at birth, 22, 33, and 44 years of age, NCDS-58 cohort

|  | **Dead (complete)** | | |  | | **Alive (complete)** | |  | **Alive (imputed)** | |
| --- | --- | --- | --- | --- | --- | --- | --- | --- | --- | --- |
|  |  | N = 1,286 |  | |  | | N = 17,272 |  |  | N = 17,272 |
|  | miss | n (%) |  | | miss | | n (%) |  | % | 95%CI |
| **At birth** |  |  |  | |  | |  |  |  |  |
| Born male | 3 | 752 (58.6) |  | | 1 | | 8,843 (51.2) |  | 51.2 | [50.5 to 52.0] |
| Short education of mother | 33 | 1,006 (80.3) |  | | 1,172 | | 12,010 (74.6) |  | 74.5 | [73.8 to 75.2] |
| Manual social class of other parent | 112 | 915 (77.9) |  | | 1,983 | | 11,077 (72.5) |  | 72.5 | [71.7 to 73.3] |
| **Cultural capital** |  |  |  | |  | |  |  |  |  |
| Less than O level at 23 | 1,063 | 102 (45.7) |  | | 4,975 | | 4,778 (38.9) |  | 39.3 | [38.4 to 40.2] |
| No numeracy problems at 23 | 1,064 | 204 (91.9) |  | | 5,005 | | 11,629 (94.8) |  | 94.6 | [94.2 to 95.1] |
| Literacy problems at 23 | 1,063 | 23 (10.3) |  | | 4,999 | | 1,176 (9.6) |  | 9.8 | [9.3 to 10.5] |
| Does not often read at 23 | 1,063 | 84 (37.7) |  | | 4,980 | | 4,782 (38.9) |  | 39.2 | [38.2 to 40.1] |
| Driver’s license at 33 | 1,150 | 104 (76.5) |  | | 6,131 | | 9,713 (87.2) |  | 86.8 | [86.1 to 87.5] |
| **Economic capital** |  |  |  | |  | |  |  |  |  |
| Personal savings > median at 23 | 1,061 | 84 (37.3) |  | | 4,961 | | 6,190 (50.3) |  | 50.2 | [49.3 to 50.7] |
| Paid work at 23 | 1,151 | 96 (71.1) |  | | 6,067 | | 8,908 (79.5) |  | 79.6 | [78.7 to 80.4] |
| Manual social class at 33 | 1,158 | 63 (49.2) |  | | 6,818 | | 4,193 (40.1) |  | 42.3 | [41.3 to 43.3] |
| **Social capital** |  |  |  | |  | |  |  |  |  |
| Does not often see friend at 23 | 1,063 | 62 (27.8) |  | | 4,976 | | 3,877 (31.5) |  | 31.7 | [30.7 to 32.6] |
| Not married at 23 | 1,061 | 128 (56.9) |  | | 4,965 | | 6,816 (55.4) |  | 55.7 | [54.7 to 56.7] |
| No child at 23 | 1,061 | 164 (72.9) |  | | 4,963 | | 9,163 (74.4) |  | 74.5 | [73.6 to 75.3] |
| Does not do laundry at 33 | 1,195 | 40 (44.0) |  | | 8,539 | | 3,803 (43.5) |  | 46.1 | [45.2 to 47.1] |
| Not religious at 23 | 1,064 | 90 (40.5) |  | | 4,976 | | 5,036 (41.0) |  | 41.3 | [40.4 to 42.3] |
| **Behaviours** |  |  |  | |  | |  |  |  |  |
| Often practices sport at 23 | 1,063 | 87 (39.0) |  | | 4,977 | | 5,858 (47.6) |  | 47.8 | [46.7 to 48.7] |
| Does not practice sport at 33 | 1,151 | 102 (75.6) |  | | 6,076 | | 8,730 (78.0) |  | 77.6 | [76.7 to 78.5] |
| Often eats fried food at 33 | 1,149 | 71 (51.8) |  | | 6,042 | | 5,068 (45.1) |  | 46.1 | [45.1 to 47.2] |
| Alcohol every day at 23 | 1,064 | 37 (16.7) |  | | 4,970 | | 2,527 (20.5) |  | 20.9 | [20.1 to 21.7] |
| Alcohol every day at 33 | 1,150 | 19 (14.0) |  | | 6,041 | | 1,381 (12.3) |  | 12.4 | [11.8 to 13.1] |
| Smoking at 23 | 1,063 | 114 (51.1) |  | | 4,967 | | 4,746 (38.6) |  | 39.1 | [38.2 to 40.0] |
| Smoking at 33 | 1,149 | 56 (40.9) |  | | 6,055 | | 3,532 (31.5) |  | 32.3 | [31.3 to 33.3] |
| Accident between 23 and 33 | 1,149 | 49 (35.8) |  | | 6,068 | | 4,617 (41.2) |  | 41.9 | [40.9 to 43.0] |
| **Biomarkers*** |  |  |  | |  | |  |  |  |  |
| Systolic Blood Pressure (mmHg) | 1,286 | -- |  | | 9,229 | | 126.5 (16.5) |  | 126.9 (16.5) | [126.5 to 127.3] |
| Log (Triglycerides (g/L)) | 1,286 | -- |  | | 7,767 | | 0.52 (0.6) |  | 0.54 (0.6) | [0.51 to 0.56] |
| LDL Cholesterol (**mmol**/L) | 1,286 | -- |  | | 7,362 | | 3.42 (0.9) |  | 3.46 (0.9) | [3.43 to 3.50] |
| HbA1c (%) | 1,286 | -- |  | | 7,893 | | 5.26 (0.7) |  | 5.27 (0.7) | [5.25 to 5.30] |
| Log (CRP (mg/L)) | 1,286 | -- |  | | 7,667 | | 0.03 (1.2) |  | 0.06 (1.2) | [0.02 to 0.10] |
| Log (Cortisol (µg)) | 1,286 | -- |  | | 6,389 | | 2.92 (0.5) |  | 2.92 (0.5) | [2.91 to 2.94] |

*Confidence intervals, computed in the 1,000 imputed bootstrapped databases; * quantitative variables, mean(sd) is given*

Table b. Level of reference for mediation analysis

| **Socio-behavioural characteristics** | **Fixed at** |
| --- | --- |
| More than O level at 23 | Yes |
| Numeracy problems at 23 | No |
| Literacy problems at 23 | No |
| Often read at 23 | Yes |
| Driver’s license at 33 | Yes |
| Personal savings > median at 23 | Yes |
| Paid work at 23 | Yes |
| Manual social class at 33 | No |
| Often see friend at 23 | Yes |
| Married at 23 | Yes |
| Child at 23 | Yes |
| Do laundry at 33 | Yes |
| Religious at 23 | Yes |
| Often practices sport at 23 | Yes |
| Does not practice sport at 33 | Yes |
| Often eats fried food at 33 | No |
| Alcohol every day at 23 | No |
| Alcohol every day at 33 | No |
| Smoking at 23 | No |
| Smoking at 33 | No |
| Accident between 23 and 33 | No |

Table c. Total effect (TE) of being born deprived on biomarkers at 44-45, NCDS-58 cohort (N = 17,272)

|  | **Original scale** | |  |  | **Z-scores** |
| --- | --- | --- | --- | --- | --- |
|  | **TE** | **95%CI** |  | **TE** | **95%CI** |
| **Systolic Blood Pressure (mmHg)** | +1.75 | [1.02 to 2.52] |  | +0.11 | [0.06 to 0.15] |
| **Log (Triglycerides (g/L))** | +0.10 | [0.06 to 0.13] |  | +0.16 | [0.11 to 0.21] |
| **LDL Cholesterol (mmol /L)** | +0.08 | [0.03 to 0.14] |  | +0.09 | [0.04 to 0.14] |
| **HbA1c (%)** | +0.08 | [0.05 to 0.11] |  | +0.10 | [0.06 to 0.15] |
| **Log (CRP (mg/L))** | +0.24 | [0.16 to 0.32] |  | +0.20 | [0.13 to 0.26] |
| **Log (Cortisol (µg))** | +0.02 | [-0.01 to 0.05] |  | +0.04 | [-0.02 to 0.10] |

*TE = total effect; Original scale = scale given with the variable name; 95%CI = bootstrapped confidence intervals (N=1,000)*

## Sensitivity analysis

Table d. Description of populations at birth, 22, 33, and 44 years of age, NCDS-58 cohort – sensitivity analysis

|  | **Non-included** | | |  | | **Included** | |  | **Included and imputed** | |
| --- | --- | --- | --- | --- | --- | --- | --- | --- | --- | --- |
|  |  | N = 11,537 |  | |  | | N = 7,021 |  |  | N = 7,021 |
|  | miss | n (%) |  | | miss | | n (%) |  | % | 95%CI |
| **At birth** |  |  |  | |  | |  |  |  |  |
| Born male | 4 | 6211 (53.9) |  | | 0 | | 3384 (48.2) |  | 48.2 | [47.0 to 49.4] |
| Short education of mother | 1,189 | 7900 (76.3) |  | | 16 | | 5116 (73.0) |  | 73.0 | [71.9 to 74.0] |
| Manual social class of other parent | 1,802 | 7294 (74.9) |  | | 293 | | 4698 (69.8) |  | 69.9 | [68.8 to 71.1] |
| **Cultural capital** |  |  |  | |  | |  |  |  |  |
| Less than O level at 23 | 6,033 | 2518 (45.7) |  | | 5 | | 2362 (33.7) |  | 33.7 | [32.6 to 34.8] |
| No numeracy problems at 23 | 6,051 | 5104 (93.0) |  | | 18 | | 6729 (96.1) |  | 96.1 | [95.6 to 96.6] |
| Literacy problems at 23 | 6,045 | 603 (11.0) |  | | 17 | | 596 (8.5) |  | 8.5 | [7.9 to 9.2] |
| Does not often read at 23 | 6,031 | 2325 (42.2) |  | | 12 | | 2541 (36.3) |  | 36.3 | [35.0 to 37.4] |
| Driver’s license at 33 | 7,186 | 3578 (82.2) |  | | 95 | | 6239 (90.1) |  | 90.1 | [89.4 to 90.8] |
| **Economic capital** |  |  |  | |  | |  |  |  |  |
| Personal savings > median at 23 | 6,022 | 2463 (44.7) |  | | 0 | | 3811 (54.3) |  | 54.3 | [53.1 to 55.4] |
| Paid work at 23 | 7,160 | 3296 (75.3) |  | | 58 | | 5708 (82.0) |  | 82.0 | [81.2 to 82.9] |
| Manual social class at 33 | 7,510 | 1747 (43.4) |  | | 466 | | 2509 (38.3) |  | 38.8 | [37.6 to 40.0] |
| **Social capital** |  |  |  | |  | |  |  |  |  |
| Does not often see friend at 23 | 6,037 | 1787 (32.5) |  | | 2 | | 2152 (30.7) |  | 30.6 | [29.7 to 31.7] |
| Not married at 23 | 6,025 | 3110 (56.4) |  | | 1 | | 3834 (54.6) |  | 54.6 | [53.5 to 55.7] |
| No child at 23 | 6,023 | 3926 (71.2) |  | | 1 | | 5401 (76.9) |  | 76.9 | [76.0 to 78.0] |
| Does not do laundry at 33 | 8,324 | 1414 (44.0) |  | | 1,410 | | 2429 (43.3) |  | 44.2 | [42.9 to 45.5] |
| Not religious at 23 | 6,035 | 2388 (43.4) |  | | 5 | | 2738 (39.0) |  | 39.0 | [37.9 to 40.3] |
| **Behaviours** |  |  |  | |  | |  |  |  |  |
| Often practices sport at 23 | 6,033 | 2473 (44.9) |  | | 7 | | 3472 (49.5) |  | 49.5 | [48.3 to 50.7] |
| Does not practice sport at 33 | 7,167 | 3316 (75.9) |  | | 60 | | 5516 (79.2) |  | 79.3 | [78.3 to 80.2] |
| Often eats fried food at 33 | 7,150 | 2143 (48.8) |  | | 41 | | 2996 (42.9) |  | 42.9 | [41.7 to 44.0] |
| Alcohol every day at 23 | 6,031 | 1087 (19.7) |  | | 3 | | 1477 (21.0) |  | 21.1 | [20.1 to 22.0] |
| Alcohol every day at 33 | 7,152 | 562 (12.8) |  | | 39 | | 838 (12.0) |  | 12.0 | [11.3 to 12.8] |
| Smoking at 23 | 6,029 | 2359 (42.8) |  | | 1 | | 2501 (35.6) |  | 35.6 | [34.5 to 36.7] |
| Smoking at 33 | 7,153 | 1600 (36.5) |  | | 51 | | 1988 (28.5) |  | 28.5 | [27.3 to 29.5] |
| Accident between 23 and 33 | 7,159 | 1752 (40.0) |  | | 58 | | 2914 (41.8) |  | 41.8 | [40.7 to 43.0] |
| **Biomarkers*** |  |  |  | |  | |  |  |  |  |
| Systolic Blood Pressure (mmHg) | 9,222 | 126.0 (16.3) |  | | 107 | | 126.5 (16.6) |  | 126.5 (16.6) | [126.1 to 126.9] |
| Log (Triglycerides (g/L)) | 9,625 | 0.55 (0.6) |  | | 1,166 | | 0.52 (0.6) |  | 0.52 (0.6) | [0.50 to 0.53] |
| LDL Cholesterol (**mmol** /L) | 9,751 | 3.44 (0.9) |  | | 1,445 | | 3.42 (0.9) |  | 3.45 (0.9) | [3.41 to 3.48] |
| HbA1c (%) | 9,587 | 5.30 (0.8) |  | | 1,068 | | 5.24 (0.7) |  | 5.25 (0.7) | [5.22 to 5.27] |
| Log (CRP (mg/L)) | 9,645 | 0.07 (1.2) |  | | 1,246 | | 0.02 (1.2) |  | 0.03 (1.2) | [-0.00 to 0.06] |
| Log (Cortisol (µg)) | 10,046 | 2.92 (0.5) |  | | 2,123 | | 2.92 (0.5) |  | 2.92 (0.5) | [2.91 to 2.94] |

*Confidence intervals, computed in the 1,000 imputed bootstrapped databases; * quantitative variables, mean(sd) is given*

Table e. Distribution of social characteristics at 23 and 33 by sex – sensitivity analysis (N=7,021)

|  | **Male-born** | |  | **Female-born** | |  | **M – F** |
| --- | --- | --- | --- | --- | --- | --- | --- |
|  | % | 95%CI |  | % | 95%CI |  | % |
| **Cultural capital** |  |  |  |  |  |  |  |
| Less than O level at 23 | 36.6 | [35.0 to 38.3] |  | 30.9 | [29.4 to 32.4] |  | +5.7 |
| No numeracy problems at 23 | 96.3 | [95.7 to 96.9] |  | 95.8 | [95.1 to 96.5] |  | +0.5 |
| Literacy problems at 23 | 10.9 | [9.8 to 11.8] |  | 6.3 | [5.5 to 7.1] |  | +4.6 |
| Does not often read at 23 | 43.9 | [42.2 to 45.7] |  | 29.1 | [27.5 to 30.5] |  | +14.8 |
| Driver’s license at 33 | 94.8 | [94.1 to 95.6] |  | 85.7 | [84.5 to 86.8] |  | +9.1 |
| **Economic capital** |  |  |  |  |  |  |  |
| Personal savings > median at 23 | 60.1 | [58.5 to 61.7] |  | 48.8 | [47.3 to 50.4] |  | +11.3 |
| Paid work at 23 | 93.1 | [92.3 to 93.9] |  | 71.7 | [70.3 to 73.1] |  | +21.4 |
| Manual social class at 33 | 47.3 | [45.4 to 49.1] |  | 30.9 | [29.3 to 32.4] |  | +16.4 |
| **Social capital** |  |  |  |  |  |  |  |
| Does not often see friend at 23 | 33.8 | [32.4 to 35.5] |  | 27.7 | [26.3 to 29.1] |  | +6.1 |
| Not married at 23 | 65.3 | [63.7 to 66.9] |  | 44.7 | [43.1 to 46.3] |  | +20.6 |
| No child at 23 | 85.0 | [83.8 to 86.3] |  | 69.5 | [68.0 to 71.0] |  | +15.5 |
| Does not do laundry at 33 | 87.0 | [85.6 to 88.4] |  | 4.3 | [3.5 to 5.1] |  | +82.7 |
| Not religious at 23 | 47.8 | [46.1 to 49.5] |  | 30.9 | [29.3 to 32.4] |  | +16.9 |
| **Behaviours** |  |  |  |  |  |  |  |
| Smoking at 23 | 36.0 | [34.3 to 37.5] |  | 35.3 | [33.7 to 37.0] |  | +0.7 |
| Smoking at 33 | 28.4 | [26.9 to 29.9] |  | 28.6 | [27.0 to 30.1] |  | -0.2 |
| Alcohol every day at 23 | 32.7 | [31.1 to 34.2] |  | 10.2 | [9.3 to 11.3] |  | +22.5 |
| Alcohol every day at 33 | 17.1 | [15.8 to 18.3] |  | 7.3 | [6.4 to 8.1] |  | +9.8 |
| Often eats fried food at 33 | 54.7 | [53.0 to 56.3] |  | 31.9 | [30.3 to 33.4] |  | +22.8 |
| Often practices sport at 23 | 61.5 | [60.0 to 63.1] |  | 38.4 | [36.8 to 39.9] |  | +23.1 |
| Does not practice sport at 33 | 80.4 | [79.0 to 81.7] |  | 78.3 | [77.0 to 79.5] |  | +2.1 |
| Accident between 23 and 33 | 59.9 | [58.2 to 61.6] |  | 25.0 | [23.6 to 26.5] |  | +34.9 |
| **Gender scores, total population** |  |  |  |  |  |  |  |
| Complete set* | 0.88 | [0.87 to 0.89] |  | 0.13 | [0.12 to 0.14] |  | +0.75 |
| Behavioural set* | 0.64 | [0.63 to 0.65] |  | 0.39 | [0.37 to 0.4] |  | +0.25 |
| Small set* | 0.59 | [0.57 to 0.6] |  | 0.44 | [0.43 to 0.46] |  | +0.15 |
| **Gender scores, in deprived-born group** |  |  |  |  |  |  |  |
| Complete set* | 0.89 | [0.88 to 0.9] |  | 0.12 | [0.11 to 0.13] |  | +0.77 |
| Behavioural set* | 0.65 | [0.64 to 0.67] |  | 0.38 | [0.37 to 0.4] |  | +0.27 |
| Small set* | 0.59 | [0.57 to 0.6] |  | 0.43 | [0.42 to 0.45] |  | +0.16 |
| **Gender scores, in advantaged-born group** |  |  |  |  |  |  |  |
| Complete set* | 0.86 | [0.85 to 0.88] |  | 0.14 | [0.12 to 0.15] |  | +0.72 |
| Behavioural set* | 0.61 | [0.6 to 0.63] |  | 0.4 | [0.38 to 0.41] |  | +0.21 |
| Small set* | 0.59 | [0.57 to 0.6] |  | 0.46 | [0.45 to 0.48] |  | +0.13 |

*Showing variable categories that were the most frequent among male-born. All categorical variables were binary.*

*“95%CI” corresponds to the confidence intervals computed on 1,000 bootstrapped imputed datasets.*

*“M-F” corresponds to the male to female differences of observed probabilities (%)*

** = quantitative variables, mean and 95%CI of mean are given*

Table f. Total effect (TE) of being born male on biomarkers at 44-45 – sensitivity analysis (N=7,021)

|  | **Female** |  | **Original scale** | |  | **Z-scores** | |
| --- | --- | --- | --- | --- | --- | --- | --- |
|  | **Mean (sd)** |  | **TE** | **95%CI** |  | **TE** | **95%CI** |
| **Systolic Blood Pressure (mmHg)** | 120.5 (15.7) |  | +12.61 | [11.86 to 13.32] |  | +0.76 | [0.72 to 0.80] |
| **Log (Triglycerides (g/L))** | 0.3 (0.5) |  | +0.42 | [0.39 to 0.45] |  | +0.71 | [0.66 to 0.76] |
| **LDL Cholesterol (mmol /L)** | 3.3 (0.9) |  | +0.33 | [0.27 to 0.40] |  | +0.36 | [0.30 to 0.42] |
| **HbA1c (%)** | 5.2 (0.6) |  | +0.13 | [0.09 to 0.16] |  | +0.19 | [0.14 to 0.24] |
| **Log (CRP (mg/L))** | 0.1 (1.3) |  | -0.09 | [-0.16 to -0.03] |  | -0.08 | [-0.14 to -0.02] |
| **Log (Cortisol (µg))** | 2.9 (0.5) |  | -0.04 | [-0.08 to -0.00] |  | -0.07 | [-0.14 to -0.01] |

*TE = total effect* *of being born male rather than female; 95%CI = bootstrapped confidence intervals (N=1,000)*

Table g. Total effect (TE) of being born deprived on biomarkers at 44-45 – sensitivity analysis (N=7,021)

|  | **Original scale** | |  |  | **Z-scores** |
| --- | --- | --- | --- | --- | --- |
|  | **TE** | **95%CI** |  | **TE** | **95%CI** |
| **Systolic Blood Pressure (mmHg)** | +1.57 | [0.82 to 2.30] |  | +0.09 | [0.05 to 0.14] |
| **Log (Triglycerides (g/L))** | +0.09 | [0.06 to 0.12] |  | +0.15 | [0.10 to 0.20] |
| **LDL Cholesterol (mmol /L)** | +0.09 | [0.04 to 0.15] |  | +0.10 | [0.04 to 0.16] |
| **HbA1c (%)** | +0.06 | [0.02 to 0.09] |  | +0.08 | [0.03 to 0.13] |
| **Log (CRP (mg/L))** | +0.24 | [0.18 to 0.31] |  | +0.20 | [0.15 to 0.26] |
| **Log (Cortisol (µg))** | +0.02 | [-0.01 to 0.05] |  | +0.04 | [-0.02 to 0.10] |

*TE = total effect; Original scale = scale given with the variable name; 95%CI = bootstrapped confidence intervals (N=1,000)*

Table h. Eliminated proportion (EP) of sex effect – sensitivity analysis (N=7,021)

|  | **(a) By gender-score mediator** | |  | **(b) By socio-behavioural mediators** | |  | **(c) By early social environment** | |  |
| --- | --- | --- | --- | --- | --- | --- | --- | --- | --- |
|  | **EP (%)** | **95%CI** |  | **EP (%)** | **95%CI** |  | **EP (%)** | **95%CI** |  |
| **Systolic Blood Pressure** |  |  |  |  |  |  | -1.14 | [-5.4 to 7.9] |  |
| Complete set | 11.06 | [-1.1 to 21.8] |  | 11.02 | [-0.6 to 21.9] |  |  |  |  |
| Behavioural set | 1.54 | [-1.9 to 3.8] |  | 1.48 | [-2.0 to 4.7] |  |  |  |  |
| Small set | 3.41 | [1.0 to 5.8] | * | 3.26 | [0.8 to 5.7] | * |  |  |  |
| **Log (Triglycerides)** |  |  |  |  |  |  | -10.23 | [-17.9 to -2.9] | * |
| Complete set | -5.13 | [-19.8 to 7.8] |  | -8.99 | [-23.8 to 4.3] |  |  |  |  |
| Behavioural set | -0.49 | [-4.9 to 3.8] |  | -0.66 | [-5.1 to 3.7] |  |  |  |  |
| Small set | -0.13 | [-3.4 to 3.0] |  | 0.08 | [-3.3 to 3.3] |  |  |  |  |
| **LDL Cholesterol** |  |  |  |  |  |  | -15.23 | [-32.0 to 0.1] |  |
| Complete set | -3.27 | [-36.8 to 29.3] |  | -13.46 | [-47.8 to 17.8] |  |  |  |  |
| Behavioural set | -1.88 | [-10.9 to 7.3] |  | -1.53 | [-10.8 to 7.6] |  |  |  |  |
| Small set | -3.90 | [-11.0 to 2.6] |  | -3.62 | [-10.8 to 3.1] |  |  |  |  |
| **HbA1c** |  |  |  |  |  |  | -3.76 | [-34.6 to 26.9] |  |
| Complete set | 15.52 | [-39.9 to 69.8] |  | -7.21 | [-67.1 to 48.0] |  |  |  |  |
| Behavioural set | -5.01 | [-22.9 to 11.5] |  | -7.49 | [-25.7 to 8.5] |  |  |  |  |
| Small set | 4.35 | [-6.8 to 17.4] |  | 4.58 | [-6.7 to 17.7] |  |  |  |  |
| **Log (CRP)** |  |  |  |  |  |  | 142.92 | [49.58 to 416.1] | * |
| Complete set | -30.21 | [-247.5 to 138.3] |  | 12.60 | [-171.3 to 198.6] |  |  |  |  |
| Behavioural set | -17.25 | [-97.4 to 29.3] |  | -16.31 | [-95.5 to 30.8] |  |  |  |  |
| Small set | -23.21 | [-95.4 to 8.4] |  | -22.49 | [-90.5 to 10.4] |  |  |  |  |
| **Log (Cortisol)** |  |  |  |  |  |  | -31.1 | [-195.6 to 83.1] |  |
| Complete set | 187.57 | [-81.7 to 774.4] |  | 184.74 | [-74.2 to 797.1] |  |  |  |  |
| Behavioural set | 21.36 | [-63.7 to 146.8] |  | 23.12 | [-57.3 to 154.4] |  |  |  |  |
| Small set | -2.21 | [-79.7 to 59.7] |  | -0.76 | [-79.1 to 66.7] |  |  |  |  |

*EP = Eliminated proportion; 95%CI = bootstrapped confidence intervals (N=1,000)*

Table i. Effects of sex and early-life social environment on biomarkers, NCDS-58 cohort (N=7,021)

|  |  | **Advantaged- born** | |  | **Deprived-born** | |  | **TE of early deprivation** | |
| --- | --- | --- | --- | --- | --- | --- | --- | --- | --- |
| **SBP** | **Original scale** |  |  |  |  |  |  |  |  |
|  | Male born (mean) | 132.1 | [131.3 to 132.8] |  | 133.8 | [133.1 to 134.5] |  | +1.7 | [0.7 to 2.7] |
|  | Female born (mean) | 119.6 | [118.9 to 120.4] |  | 121.1 | [120.3 to 121.8] |  | +1.5 | [0.5 to 2.5] |
|  | TE of being born male | +12.5 | [11.4 to 13.5] |  | +12.7 | [11.7 to 13.7] |  | **(0.25)** | **[-1.17 to 1.69]** |
|  | **Z-score** |  |  |  |  |  |  |  |  |
|  | Male born (mean) | 0.33 | [0.29 to 0.38] |  | 0.44 | [0.4 to 0.47] |  | +0.10 | [0.04 to 0.16] |
|  | Female born (mean) | -0.42 | [-0.46 to -0.38] |  | -0.33 | [-0.36 to -0.29] |  | +0.09 | [0.03 to 0.15] |
|  | TE of being born male | +0.75 | [0.69 to 0.81] |  | +0.77 | [0.71 to 0.82] |  | **(0.02)** | **[-0.07 to 0.10]** |
| **Log(triglycerides)** | **Original scale** |  |  |  |  |  |  |  |  |
|  | Male born (mean) | 0.71 | [0.67 to 0.74] |  | 0.75 | [0.72 to 0.78] |  | +0.05 | [0.00 to 0.09] |
|  | Female born (mean) | 0.24 | [0.21 to 0.27] |  | 0.36 | [0.34 to 0.39] |  | +0.12 | [0.08 to 0.16] |
|  | TE of being born male | +0.46 | [0.42 to 0.51] |  | +0.39 | [0.35 to 0.43] |  | **(-0.09)** | **[-0.17 to -0.00]** |
|  | **Z-score** |  |  |  |  |  |  |  |  |
|  | Male born (mean) | 0.32 | [0.27 to 0.37] |  | 0.40 | [0.36 to 0.44] |  | +0.08 | [0.01 to 0.16] |
|  | Female born (mean) | -0.47 | [-0.51 to -0.42] |  | -0.26 | [-0.29 to -0.22] |  | +0.21 | [0.15 to 0.27] |
|  | TE of being born male | +0.79 | [0.71 to 0.86] |  | +0.66 | [0.59 to 0.72] |  | **(-0.09)** | **[-0.19 to -0.00]** |
| **LDL cholesterol** | **Original scale** |  |  |  |  |  |  |  |  |
|  | Male born (mean) | 3.59 | [3.53 to 3.65] |  | 3.64 | [3.57 to 3.72] |  | +0.05 | [-0.02 to 0.13] |
|  | Female born (mean) | 3.21 | [3.16 to 3.26] |  | 3.34 | [3.30 to 3.38] |  | +0.14 | [0.08 to 0.20] |
|  | TE of being born male | +0.38 | [0.31 to 0.46] |  | +0.30 | [0.22 to 0.38] |  | **(-0.08)** | **[-0.13 to -0.02]** |
|  | **Z-score** |  |  |  |  |  |  |  |  |
|  | Male born (mean) | 0.15 | [0.1 to 0.21] |  | 0.21 | [0.16 to 0.26] |  | +0.05 | [-0.03 to 0.14] |
|  | Female born (mean) | -0.26 | [-0.31 to -0.21] |  | -0.11 | [-0.15 to -0.07] |  | +0.15 | [0.08 to 0.21] |
|  | TE of being born male | +0.41 | [0.33 to 0.48] |  | +0.32 | [0.24 to 0.40] |  | **(-0.13)** | **[-0.22 to -0.04]** |
| **HbA1c** | **Original scale** |  |  |  |  |  |  |  |  |
|  | Male born (mean) | 5.28 | [5.25 to 5.32] |  | 5.33 | [5.30 to 5.38] |  | +0.05 | [0.00 to 0.10] |
|  | Female born (mean) | 5.15 | [5.12 to 5.18] |  | 5.21 | [5.18 to 5.24] |  | +0.06 | [0.02 to 0.10] |
|  | TE of being born male | +0.13 | [0.09 to 0.18] |  | +0.12 | [0.08 to 0.18] |  | **(-0.01)** | **[-0.07 to 0.06]** |
|  | **Z-score** |  |  |  |  |  |  |  |  |
|  | Male born (mean) | 0.05 | [0.01 to 0.10] |  | 0.13 | [0.08 to 0.17] |  | +0.08 | [0.00 to 0.15] |
|  | Female born (mean) | -0.14 | [-0.18 to -0.10] |  | -0.05 | [-0.09 to -0.02] |  | +0.09 | [0.03 to 0.14] |
|  | TE of being born male | +0.20 | [0.13 to 0.26] |  | +0.18 | [0.11 to 0.25] |  | **(-0.01)** | **[-0.11 to 0.08]** |
| **Log (CRP)** | **Original scale** |  |  |  |  |  |  |  |  |
|  | Male born (mean) | -0.10 | [-0.16 to -0.03] |  | 0.04 | [-0.02 to 0.09] |  | +0.13 | [0.05 to 0.22] |
|  | Female born (mean) | -0.12 | [-0.20 to -0.04] |  | 0.22 | [0.16 to 0.28] |  | +0.34 | [0.25 to 0.43] |
|  | TE of being born male | +0.03 | [-0.07 to 0.12] |  | -0.18 | [-0.26 to -0.10] |  | **(-0.21)** | **[-0.32 to -0.09]** |
|  | **Z-score** |  |  |  |  |  |  |  |  |
|  | Male born (mean) | -0.11 | [-0.15 to -0.06] |  | 0.01 | [-0.03 to 0.05] |  | +0.11 | [0.04 to 0.18] |
|  | Female born (mean) | -0.13 | [-0.18 to -0.07] |  | 0.16 | [0.12 to 0.20] |  | +0.28 | [0.21 to 0.36] |
|  | TE of being born male | +0.02 | [-0.06 to 0.10] |  | -0.15 | [-0.22 to -0.08] |  | **(-0.17)** | **[-0.27 to -0.08]** |
| **Log (Cortisol)** | **Original scale** |  |  |  |  |  |  |  |  |
|  | Male born (mean) | 2.89 | [2.85 to 2.92] |  | 2.91 | [2.88 to 2.95] |  | +0.03 | [-0.01 to 0.07] |
|  | Female born (mean) | 2.93 | [2.90 to 2.97] |  | 2.95 | [2.92 to 2.98] |  | +0.01 | [-0.02 to 0.05] |
|  | TE of being born male | -0.05 | [-0.09 to 0.00] |  | -0.03 | [-0.07 to 0.01] |  | **(+0.01)** | **[-0.04 to 0.07]** |
|  | **Z-score** |  |  |  |  |  |  |  |  |
|  | Male born (mean) | -0.07 | [-0.13 to -0.01] |  | -0.02 | [-0.06 to 0.03] |  | +0.05 | [-0.03 to 0.13] |
|  | Female born (mean) | 0.02 | [-0.03 to 0.07] |  | 0.05 | [0.00 to 0.09] |  | +0.03 | [-0.05 to 0.10] |
|  | TE of being born male | -0.09 | [-0.17 to 0.00] |  | -0.06 | [-0.14 to 0.02] |  | **(+0.03)** | **[-0.07 to 0.12]** |

*SBP = Systolic Blood Pressure; TE = Total effect; 95%CI = bootstrapped confidence intervals (N=1,000); results in bold and in brackets are the measures of additive interaction* ${(Y}_{11}-Y_{10})-\left( Y_{01}-Y_{00} \right)$
